# Optimization Techniques for Permittivity and Permeability Determination

**DOI:** 10.6028/jres.096.033

**Published:** 1991

**Authors:** Paul D. Domich, James Baker-Jarvis, Richard G. Geyer

**Affiliations:** National Institute of Standards and Technology, Boulder, CO 80303

**Keywords:** higher order modes, microwave, permeability, permittivity, primary mode, orthogonal distance regression, scattering matrix

## Abstract

This paper discusses optimization techniques for the determination of complex permittivity and permeability in transmission lines. The traditional theoretical model using scattering parameters is extended into a mathematical regression model that can be solved with widely accepted numerical techniques. This new model produces accurate primary mode results for the samples tested including nonmagnetic and magnetic materials with high dielectric constants. An extension of the model includes responses due to higher order modes. The general model determines parameters to specify the spectral functional form of complex permittivity and permeability and is capable of small corrections to independent variable data including angular frequency, sample length, sample position, and cutoff wavelength. The method provides reliable determination for both low and high permittivity materials.

## 1. Introduction

A constrained nonlinear optimization procedure is presented for the determination of complex permittivity and permeability spectra from scattering parameter (*S*-parameter) data taken from an automatic network analyzer (ANA). The procedure has been used successfully for reliable characterization of permittivity and permeability of many different test samples. In addition, it provides a basis for the analysis of multi-mode field data and for the determination of experimental systematic uncertainty.

Previous work in this area involved the determination of permittivity and permeability on a point-by-point basis with explicit or implicit solution of a system of nonlinear scattering equations at each particular frequency (see [1, 5]). Inaccurate results, however, may arise when numerical singularities occur at frequencies corresponding to integer multiples of one half wavelength of the material. The same system of nonlinear scattering equations is used in this study. Here they are solved in the sense of least squares over the entire range of measurement by determining the best Laurent series approximations to permittivity and permeability consistent with linearity and causality constraints. Points of singularity may be de-emphasized to lessen the effect of highly uncertain data points.

This effort determines complex permittivity and permeability from two-port *S*-parameter data using the primary (or fundamental) mode field behavior in various materials. Physical measurements of the *S*-parameter data are made with an automatic network analyzer. Fundamental mode *S*-parameter relationships are then used to solve for permeability and permittivity, *µ*(*ω*) and *ϵ*(*ω*) respectively, as a function of the angular frequency *ω*.

The general functional form for *µ* and *ϵ* was decided upon after evaluating several different polynomial and trigonometric relations. The evaluation criteria for the functional form for permittivity and permeability was based on the reproducibility of the *S*-parameter data in terms of the minimum total least square approximation to these data. The best overall approximation was selected. The general functional form for *µ* and *ϵ* with acceptable results involves only the first two terms of a Laurent series.

The optimization approach in this research is an implicit function regression model. This model is solved by the orthogonal distance regression package ODRPACK [2]. This estimation package allows for adjustments in input parameters to compensate for measurement uncertainties. Adjustments here are limited to the sample length, sample position in the waveguide measurement fixture, and the cutoff wavelength. Orthogonal distance regression is intended to compensate for slight uncertainties in the independent variable (angular frequency, *ω*) as well as the dependent variable (observed *S*-parameter data).

The physical model is outlined in Sec. 2, with the related mathematical model discussed in Sec. 3. Numerical considerations are covered in Sec. 4 and conclusions and future directions discussed in Sec. 5.

## 2. Scattering Parameter Relations

The equations described below relate the measured two-port scattering parameters (*S*-parameters) to the permittivity and permeability of the material. First, in order to develop the scattering equations, the following notation is used. Let

$$\epsilon =\left[\epsilon_{R}^{\prime }-j\epsilon_{R}^{"}\right]\epsilon_{0}=\epsilon_{R}^{*}\epsilon_{0}$$

and

$$\mu =\left[\mu_{R}^{\prime }-j\mu_{R}^{"}\right]\mu_{0}=\mu_{R}^{*}\mu_{0},$$

the permittivity and permeability of a sample material, where *ϵ*_0_ and *µ*_0_ are the permittivity and permeability of a vacuum, and $\epsilon_{R}^{*}$ and $\mu_{R}^{*}$ are the relative complex permittivity and permeability. Next let *c*_vac_ and *c*_lab_ be the speed of light in a vacuum and the laboratory, respectively, and for a given frequency *f*, let

ω=2πf,

be the corresponding angular frequency. Then

$$\gamma =j\sqrt{\frac{\omega^{2}\mu_{R}^{*}\epsilon_{R}^{*}}{C_{\text{vac}}^{2}}-{\left(\frac{2\pi }{\lambda_{c_{1}}}\right)}^{2}}$$

and

$$\gamma_{0}=j\sqrt{{\left(\frac{\omega }{c_{\text{lab}}}\right)}^{2}-{\left(\frac{2\pi }{\lambda_{c_{1}}}\right)}^{2}}$$

represent the propagation constants in the material and air, respectively, where $j=\sqrt{-1}$ and $\lambda_{c_{1}}$ is the cutoff wavelength in the waveguide measurement fixture, where the subscript 1 refers to the fundamental mode. The expression for the transmission coefficient *z* is

z=exp(−γL),

where *L* is the sample length. The reflection coefficient is

$$\Gamma =\frac{\frac{\mu }{\mu_{0}}\frac{\gamma_{0}}{\gamma }-1}{\frac{\mu }{\mu_{0}}\frac{\gamma_{0}}{\gamma }+1}$$

or

$$\Gamma =\frac{\frac{c_{\text{vac}}}{c_{\text{lab}}}\sqrt{\frac{\mu_{R}^{*}}{\epsilon_{R}^{*}}}-1}{\frac{c_{\text{vac}}}{c_{\text{lab}}}\sqrt{\frac{\mu_{R}^{*}}{\epsilon_{R}^{*}}}+1}$$

for coaxial line when $\frac{1}{\lambda_{c_{1}}}\to 0$.

**Figure 1:**
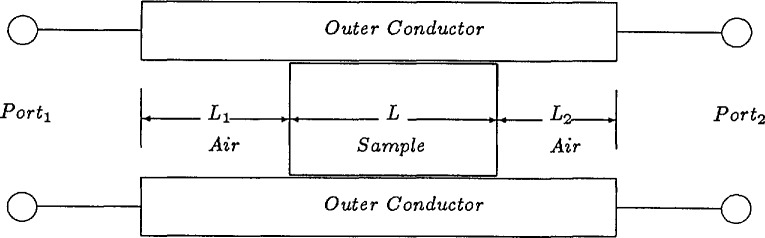
Dielectric sample in waveguide.

It is assumed that the total length of the sample holder is

$$L_{\text{air}}=L+L_{1}+L_{2},$$ (1)

as shown in Fig. 1, where *L*_1_ and *L*_2_ are the distances from the calibration reference planes to the sample faces for ports 1 and 2, respectively.

For a two-port device the expressions for the measured *S*-parameters are obtained by the solution of a related boundary value problem. The explicit expressions for the scattering relations of the fundamental mode are assumed to functions of $\lambda_{c_{1}}$ are given by

$$s_{11}(\lambda_{c_{1}})=R_{1}^{2}[\frac{\Gamma (1-z^{2})}{1-\Gamma^{2}\;z^{2}}],$$ (2)

$$s_{12}\left(\lambda_{c_{1}}\right)=R_{1}R_{2}\left[\frac{z\left(1-\Gamma^{2}\right)}{1-\Gamma^{2}\;z^{2}}\right],$$ (3)

$$s_{21}\left(\lambda_{c_{1}}\right)=R_{1}R_{2}\left[\frac{z\left(1-\Gamma^{2}\right)}{1-\Gamma^{2}\;z^{2}}\right],$$ (4)

$$s_{22}\left(\lambda_{c_{1}}\right)=R_{2}^{2}\left[\frac{\Gamma \left(1-z^{2}\right)}{1-\Gamma^{2}\;z^{2}}\right],$$ (5)

where *R*_1_ = exp(−γ_0_ *L*_1_) and *R*_2_= exp(−γ_0_ *L*_2_) are the reference plane transformation expressions.

## 3. The Mathematical Model

### 3.1 $\mu_{R}^{*}\left(\omega \right)$, $\epsilon_{R}^{*}\left(\omega \right)$, and the Regression Model

The mathematical problem can be stated as that of finding parameters to a prespecified functional form for the complex functions $\mu_{R}^{*}\left(\omega \right)$ and $\epsilon_{R}^{*}\left(\omega \right)$. *S*-parameter data acquired from the ANA for selected samples of materials provide complex values for each of *S*_11_, *S*_21_, *S*_12_, and *S*_22_, at *n* different frequencies ranging from 1 to 18 GHz. The model determines the parameterization of $\mu_{R}^{*}$ and $\epsilon_{R}^{*}$ that best reproduces simultaneously the four *S*-parameters for the *n* observations, given the sample data, two reference plane positions, and the sample length.

The general form for $\mu_{R}^{*}\left(\omega \right)$ and $\epsilon_{R}^{*}\left(\omega \right)$ uses terms from the Laurent series:

$$f\left(\omega \right)=\sum_{i=-\infty }^{\infty }\frac{a_{i}}{{\left(1+b_{i}\omega \right)}^{i}},$$ (6)

where *a_i_* and *b_i_* are complex scalars in the *i* th term. The solution procedure here is an implicit function regression and corresponds to the parameterization of two terms in a truncated Laurent series. Note that Eq. (7) automatically satisfies Kramers-Kronig relations (see, for example, [1] or [4]) for dispersion. The functional form for $\mu_{R}^{*}\left(\omega \right)$ and $\epsilon_{R}^{*}\left(\omega \right)$ follows:

$$\begin{array}{l} \mu_{R}^{*}\left(\omega \right)\equiv f\left(\omega \right)=\frac{A_{1}}{1+B_{1}\omega }\;+\frac{A_{2}}{(1+B_{2}\omega {)}^{2}},\\ \epsilon_{R}^{*}\left(\omega \right)\equiv \tilde{f}\left(\omega \right)=\frac{{\tilde{A}}_{1}}{1+{\tilde{B}}_{1}\omega }+\frac{{\tilde{A}}_{2}}{{\left(1+{\tilde{B}}_{2}\omega \right)}^{2}}. \end{array}$$

For notational convenience, the terms of the second truncated Laurent series *Ã*_1_, *Ã*_2_, ${\tilde{B}}_{1}$, and ${\tilde{B}}_{2}$ will be referred to as *A*_3_, *A*_4_, *B*_3_, and *B*_4_.

The solution procedure to determine the complex parameters *A_i_*, *B_i_* is the minimization of the sum of the squares of the uncertainties between the predicted and observed *S*-parameters,

$$\begin{array}{l} \min(\sum_{k=1}^{n}|S_{11}^{k}-P_{11}^{k}{|}^{2}+|S_{12}^{k}-P_{12}^{k}{|}^{2}+\\ {|S_{21}^{k}-P_{21}^{k}{|}^{2}+|S_{22}^{k}-P_{22}^{k}{|}^{2})}^{1/2} \end{array}$$ (7)

where, for *i*, *j* = 1, 2, $S_{ij}^{k}$ represents the *k* th observed *S_ij_* scattering parameter at frequency *ω_k_* and

$$p_{ij}^{k}\equiv S_{ij}\left(\lambda_{c_{1},}\omega_{k},A_{1},...,A_{4},B_{1},...,B_{4}\right),$$

is the corresponding predicted scattering parameter. We let $|z|=\sqrt{ℜ{\left(z\right)}^{2}+ℑ{\left(z\right)}^{2}}$ represent the absolute value of a complex scalar *z*. Note that Eq. (8) is equivalent to the minimization of the sum of the squares of the real and imaginary parts of each of the *S*-parameters, i. e.,

$$\min{\left({\sum_{k=1}^{n}\sum_{i=1}^{2}\sum_{j=1}^{2}ℜ{\left(S_{ij}^{k}-P_{ij}^{k}\right)}^{2}+ℑ\left(S_{ij}^{k}-P_{ij}^{k}\right)}^{2}\right)}^{1/2}$$ (8)

The model reported in Sec. 4.4 results uses the formulation in Eq. (8).

### 3.2 Adjustments to the Model Inputs

In standard ordinary least squares regression models, the observed responses are assumed to contain some uncertainties either produced by the phenomena under examination or introduced by the device that measures the events. In addition, certain independent and dependent variable pairs may not be as reliable as others due to an increased variance in the uncertainties in the dependent variable for particular values of the independent variable. The application of a reduced weight to points of questionable reliability provides the modeler with a means to de-emphasize such points to find a more appropriate regression solution.

Usually the independent variable can be controlled, and the precise value of each of the observations is well known. An orthogonal distance regression model provides the modeler with the additional ability to assume that the independent variable, in this case frequency, may contain some uncertainty as well. Allowances for this type of uncertainty can, in some cases, greatly improve the approximation. For this particular model and the samples tested in this study, the uncertainty in the independent variables is sufficiently small to allow the modeler to assume that an ordinary least squares approximation provides an adequate solution.

Other model parameters such as sample length, sample position in the waveguide, and cutoff wavelength are sufficiently sensitive to require slight perturbation. Each of these inputs is required by the *S*-parameter equations and is considered to be known by the modeler. Since these inputs are not always known exactly, each may be perturbed slightly, as determined by the problem solver, to improve approximation. This allows the user to adjust for measurement uncertainty.

In particular, since incorrect specification of the sample position, *L*_1_, in waveguide affects the value of phase in the reflected *S*-parameter data, an additional parameter, $\beta_{L_{1}}$, is included in *R*_1_,

$$R_{1}=\exp\left(-\gamma_{0}\left[L_{1}+\beta_{L_{1}}\right]\right).$$

Similarly, uncertainties in *L*_air_ and *L*_2_ are represented by analogous means with parameters $\beta_{L_{\text{air}}}$ and $\beta_{L_{2}}$, respectively. With Eq. (1), the total length *L* of the sample is completely determined by

$$L=\left(L_{\mathrm{air}}+\beta_{L_{\mathrm{air}}}\right)-\left(L_{1}+\beta_{L_{1}}+L_{2}+\beta_{L_{2}}\right)$$

and is parameterized by the values of $\beta_{L_{\text{air}}}$, $\beta_{L_{1}}$ and $\beta_{L_{2}}$.

Another parameterized correction, *β*_λ_, is included in the cutoff wavelength $\lambda_{c_{1}}$, as $\lambda_{c_{1}}+\beta_{\lambda }$. Inaccuracies in the waveguide dimensions due to the milling process can affect the value for the cutoff wavelength. In addition, the higher order model discussed below requires an additional wavelength cutoff value, $\lambda_{c_{2}}$, for a higher mode solution. Since it is not known *a priori* which higher modes will be present, a variation in $\lambda_{c_{2}}$ from $0<\lambda_{c_{2}}\leq \lambda_{c_{1}}+\beta_{\lambda }$, enables the solution procedure to find the value for $\lambda_{c_{2}}$ that best improves the *S*-parameter approximation for the higher order terms.

One can formulate an invariant model with respect to reference planes for the problem discussed here, as suggested in [1], to remove uncertainty in *L*_1_ and in *L*_2_. This approach uses Eq. (3) or (4), and the determinant of the *S*-matrix,

$$\det\left(S\right)=S_{11}S_{22}-S_{12}S_{21},$$

and solves for the parameters *A*_1_,…, *A*_4_, *B*_1_*…*, *B*_4_. Simplification of det(*S*) yields a formula with neither *L*_1_ nor *L*_2_. This reduced model remains dependent on a precise knowledge of both L_air_ and *L* and the cutoff wavelength, $\lambda_{c_{1}}$. This approach can be used interchangeably with the original model that contains *L*_1_ and *L*_2_. The original model uses twice the number of observations and seems to produce more accurate approximations to the *S*-parameter data.

### 3.3 Higher Order Modes

In samples with a high dielectric constant, the observed *S*-parameter data may exhibit responses due to modes other than the fundamental mode. These responses are the result of resonances of the higher mode in the material. Earlier work does not include this information in the computation of $\mu_{R}^{*}$ and $\epsilon_{R}^{*}$ as higher modes are ignored. In this section we describe an enhancement to the previously defined model that does include higher order response data.

Because of the similarity between the response of the primary mode and the higher modes (see Sec. 4.4), an additional term is added to each of the four equations (see Eqs. 2–5). This numerical model includes higher order mode structure in the *S*-parameter approximation and is a simple extension of the primary mode model described in Sec. 2.^1^ The terms that approximate the higher order modes are identical to the primary mode term with the exception that each term is scaled. For the evaluation of the higher order terms, all input parameters are unchanged except for the parameter for the higher mode cutoff wavelength, $\lambda_{c_{2}}$, that is allowed to decrease.

The explicit forms of the new set of equations use the Eqs. (2–5), and are denoted as *D*_11_, *D*_12_, *D*_21_, and *D*_22_. Associated with each higher order term is a scaling parameter, *β*_1_,…, *β*_4_. In particular, to include the primary mode and one higher-order mode, the following approximate model is used:

$$D_{11}=S_{11}\left(\lambda_{c_{1}}\right)\;+\;\beta_{1}S_{11}\left(\lambda_{c_{2}}\right),$$ (9)

$$D_{21}=S_{21}\left(\lambda_{c_{1}}\right)\;+\;\beta_{2}S_{21}\left(\lambda_{c_{2}}\right),$$ (10)

$$D_{12}=S_{12}\left(\lambda_{c_{1}}\right)\;+\;\beta_{3}S_{12}\left(\lambda_{c_{2}}\right),$$ (11)

$$D_{22}=S_{22}\left(\lambda_{c_{1}}\right)\;+\;\beta_{4}S_{22}\left(\lambda_{c_{2}}\right),$$ (12)

where $\lambda_{c_{2}}$ is determined by ODRPACK.

### 3.4 The Initial Solution Procedure

In the Nicolson-Ross-Weir procedure (5, 7) the equations for the scattering parameters are combined to allow the system of equations to decouple. This decoupling yields an explicit equation for the permittivity and permeability as a function of the scattering parameters on a point-by-point basis. This solution procedure is the basis of the commonly used techniques for obtaining permittivity and permeability. Unfortunately, these equations are not well-behaved for low-loss materials at frequencies that correspond to integer multiples of one half wavelength in the sample.

The Nicolson-Ross-Weir procedure that is implemented provides a good initial approximation to *A*_1_, *A*_3_, *B*_1_ and *B*_3_. All other parameters are initialized to zero. The estimated values for permittivity and permeability are determined on a point-by-point basis by frequency. The corresponding scattering parameters are computed with these values and then compared to the observed values. The computed values for $\mu_{R}^{*}$ and $\epsilon_{R}^{*}$ that provide the closest agreement between the observed and predicted *S*-parameter data are used as the initial values to the regression model.

## 4. Numerical Considerations

### 4.1 ODRPACK Orthogonal Distance Regression Package

Briefly, ODRPACK [2] is an implementation of a trust region Levenberg-Marquardt algorithm. This type of trust region approach adaptively determines the region in which the linear approximation closely resembles the nonlinear model. The procedure allows both an ordinary least squares model, in which the uncertainties are assumed to be only in the dependent variable, and, an orthogonal distance regression model, where uncertainties also exist in the independant variables.

First order derivatives for the Jacobian matrices can be numerically approximated (finite difference approximation), or can be user-supplied analytical derivatives. The procedure performs automatic scaling of the variables if necessary, as well as determination the precision of the model in terms of machine precision. The ODRPACK includes many other features that assist the user in the modeling process. The model automatically determines the number of digits in the model, checks analytical derivatives provided by the user, and automatically selects many of the input parameters for the user, if desired.

Iterations are stopped in ODRPACK when any one of three stopping criteria is met. Two of these indicate that the iterations have converged to a solution. Sum-of-squares convergence indicates that the change in sum-of-squares observational uncertainty is sufficiently small. Parameter convergence indicates that the change in the estimated parameters is sufficiently small. The third stopping criterion is a limit on the number of iterations.

### 4.2 Initial Conditions

Many of the input options for ORDPACK can be set to their default values, as is done in this model. The most significant input parameters for modeling permittivity and permeability are the initial values for *A*_1_, *A*_3_, *B*_1_ and *B*_3_. Sensitivity to the initial solution for these parameters is discussed below and the selection of initial settings is covered in Sec. 3.4. When higher modes are included, the solution for the primary mode is used as the initial guess for the higher mode model. For all of the parameters that define the physical model except those for $\mu_{R}^{*}$ and $\epsilon_{R}^{*}$ mentioned above, the standard laboratory values are used. All additional parameters are initialized to zero.

### 4.3 Sample Characteristics

**Figure 2:**
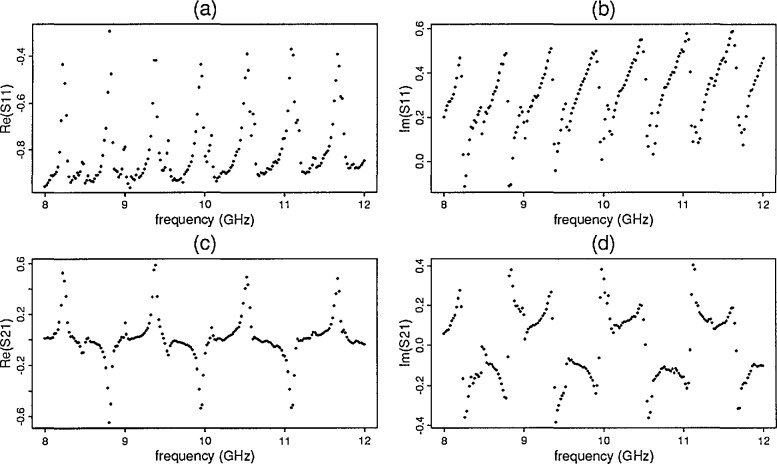
Barium titanate mix 1 (a) ℜ [*S*_11_], (b) ℑ [*S*_11_], (c) ℜ [*S*_21_], (d) ℑ [*S*_21_].

A total of seven samples were modeled to determine permittivity and permeability with ANA two-port *S*-parameter data. The sample characteristics appear in Table 1. Figure 2 contains selected plots of the observed ANA *S*-parameter data versus frequency for sample 6.

### 4.4 Numerical Results

**Fig. 3:**
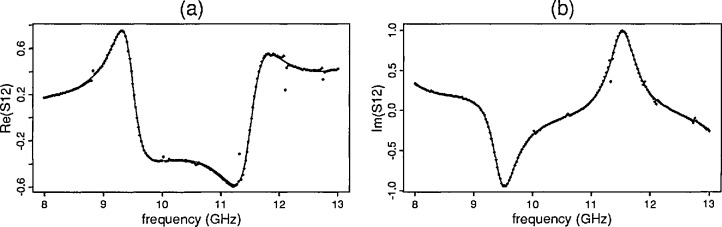
Yttrium iron garnet predicted and observed: (a) ℜ [*S*_12_] (b) ℑ [*S*_12_].

**Fig. 4:**
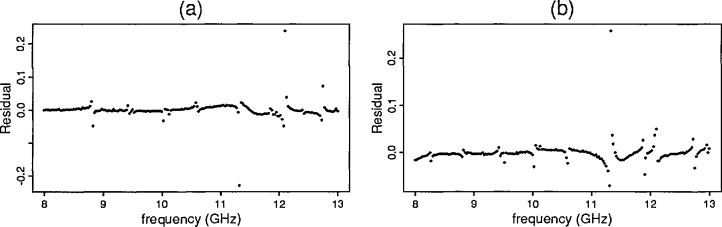
Yttrium iron garnet residual plots: (a) ℜ [*S*_12_] (b) ℑ [*S*_12_.

The first results reported are those for the primary mode model for all seven samples described in Sec. 4.3. The first set of plots (Fig. 3) include the observed *S*-parameter data (dots) for the YIG sample (sample 4) from the ANA overlaid by the predicted data (line) found by the model. The corresponding residual plots^2^ for this sample appear in Fig. 4.

**Figure 5:**
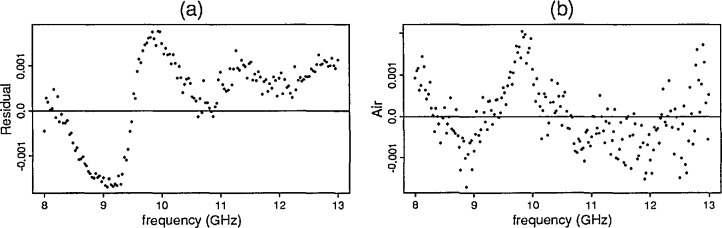
(a) Residual plots of ℑ [*S*_22_] for cross-linked polystyrene (b) ℑ [*S*_22_] for an empty waveguide.

For the first four samples the predicted and observed data are nearly identical. The residual plot for cross-linked polystyrene (sample 1) as shown in Fig. 5 reveals the systematic uncertainty due to the ANA.

**Figure 6:**
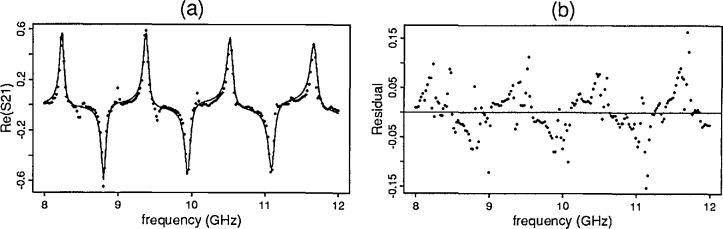
Barium titanate mix 1 (a) predicted and observed ℜ [*S*_21_] (b) residual plot.

**Figure 7:**
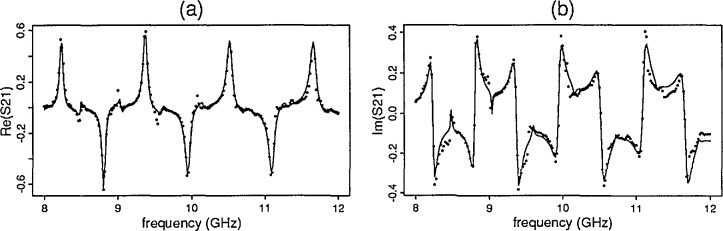
Barium titanate mix 1 (a) predicted and observed ℜ [*S*_21_] (b) predicted and observed ℜ [*S*_21_].

Fig. 6 illustrates a *S*-parameter primary mode solution for barium titanate mix 1 with a high dielectric constant and its corresponding residual plot. For three of the samples with high dielectric constants, the new model produces responses for the primary and one of the higher modes. See Fig. 7 for the *S*-parameter real and imaginary components of *S*_21_ for the sample exhibited in Fig. 6 with the model for higher order modes.

**Figure 8:**
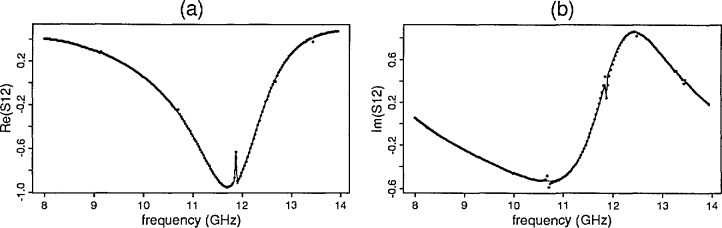
Nickel ferrite (a) predicted and observed ℜ [*S*_21_] (b) predicted and observed ℑ [*S*_21_].

For samples 5, 6, and 7 the predicted *S*-parameter data provide realistic primary mode responses although the residual plots for these samples indicate that higher modes are present. With the higher order model described in Sec. 3.3 and the new solution found, the problem is resolved for the last three samples. The new solution specifies the additional parameters *β*_1_,…, *β*_4_. Fig. 8 shows the results from the higher order model for sample 5. Additional work in this area suggests that this model is only an approximation.

### 4.5 ANA Systematic Uncertainty

The difference of the predicted *S*-parameter and the observed values for samples with low dielectric values revealed systematic uncertainty. (For the high dielectric samples, other sources of uncertainty including higher mode responses dominate the ANA-induced uncertainty.) Additional tests revealed that the source of the uncertainty is not related to the material tested in the waveguide. In fact, uncertainties in the *S*-parameter data for the cross-linked polystyrene sample closely resemble the *S*-parameter data for an empty waveguide. Attempts to further identify and remove the systematic uncertainty are underway. Figure 5 contains the *S*_11_ data for an empty waveguide and also the residual plot for the cross-linked polystyrene sample.

### 4.6 Permittivity and Permeability Estimates

The estimates of permeability and permittivity for the various samples are defined by the parameters *A*_1_, …*A*_4_, *B*_1_, … *B*_4_, as a function of frequency. Several of the samples are magnetic (see Table 1). For these samples the permeability was allowed to vary, with ODRPACK required to determine the (1,0) value. Slight variations in the value of permeability are apparent although the deviation from the true value is small. The solution procedure also provides the standard deviation and confidence intervals for each of the estimated parameters.

### 4.7 Robustness of the ODRPACK Procedure

The robustness of a mathematical procedure is its ability to find a locally optimal solution from a varied initial condition. The robustness of the entire permeability and permittivity procedure depends on the robustness of the ODRPACK procedure and, more significantly, the robustness of the mathematical model. The existence of alternative optima in the mathematical model can limit the range of the initial conditions to produce a particular solution. In addition, singularities in the absence of alternative optima may force the solution procedure to fail to determine directions of improvement and cause a premature termination.

For the samples in this study, variability in the robustness of the procedure depended on the sample studied. For the materials with small dielectric constant, the procedure readily determined the correct solution for a variety of initial conditions. For materials with higher dielectric constant, the procedure often converged quickly, although the existence of alternative optima in the mathematical model often required the repeated solution with varied initial conditions before an acceptable solution was found.

In particular, for the cross-linked polystyrene sample, initial values for $\mu_{R}^{*}$ and $\epsilon_{R}^{*}$ were set to (1,0) and (1,0) and were constant over the entire frequency range. The solution (1,0), (2.517,0.0018) was found after 50 iterations of the solution procedure. Initial conditions of (1,0), and (4.0,0.01) failed to determine a solution, while altering the imaginary part of $\epsilon_{R}^{*}$ to 1×10^−4^ resulted in the desired final solution once again. For material with high dielectric constants, the range was smaller. For example for the barium titanate mix 1, initial conditions of (1.0,0.0) and (200.0,1.0) produced the converged value of (1,0) and (269.0,1.70) in 59 iterations.

One should note that the solution procedure can change the value of the estimated parameters by large amounts in the early iterations. Hence it is possible that for cross-linked polystyrene an initial solution farther from the desired solution may in fact find a solution merely because of the solution path taken by the procedure.

## 5. Conclusions and Future Directions

**Figure 9:**
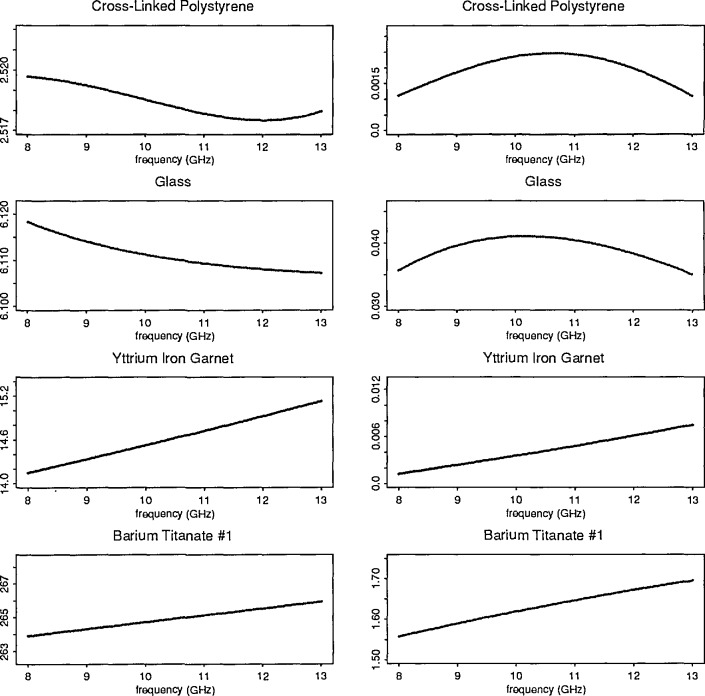
Selected plots of $\epsilon_{R}^{*}$ (*ω*) for various samples.

The nonlinear optimization procedure using two-port scattering parameters determined permittivity and permeability for a large number of samples (see Fig. 9). The added capability that permits variations in certain input parameters provides a mechanism to adjust for measurement uncertainties. The extension of the model to include higher order mode responses is quite useful for high dielectric constant materials.

## Figures and Tables

**Table 1:** Sample characteristics

	Material name	Identifier	Length (m)	*L*_1_ (m)	*L*_2_ (m)	Initial $\epsilon_{R}^{*}$	Initial $\mu_{R}^{*}$
1	Cross-linked polystyrene	rexa240889	2.407×10^−2^	0.000	1.3208×10^−3^	(2.53,0.002)	(1,0)
2	1723 glass	172a240889	1.015×10^−2^	0.000	1.5250×10^−2^	(6.15,0.04)	(1,0)
3	Loaded polymer	112a050290	2.540×10^−2^	0.000	0.0000	(5.75,0.23)	(1.6,0.1)
4	Yttrium iron garnet	YIG	1.766×10^−2^	0.000	7.7380×10^−3^	(10,0.2)	(1,0.4)
5	Nickel ferrite	tt1a120490	1.013×10^−2^	0.000	1.5263×10^−2^	(11.5,0.1)	(0.85,0.1)
6	Barium titanate mix 1	barium	7.632×10^−3^	0.000	1.7768×10^−2^	(265,1)	(1,0)
7	Barium titanate mix 2	french	2.427×10^−2^	0.000	1.1252×10^−3^	(105,1)	(1,0)

## Footnotes

1 The justification of new terms added to each of Eqs. 2–5 is based solely on empirieal evidenee found when solving for the primary mode.

2 A “residual plot” denotes a plot of the difference between the predicted and observed *S*-parameter data over the range of observations.
